# Poly[[[μ_3_-3-(2-carboxyl­atophen­yl)propionato][μ_2_-*N*,*N*′-(ethane-1,2-di­yl)bis­(pyridine-4-carbox­amide)]copper(II)] monohydrate], a layered coordination polymer with (4,4) topology

**DOI:** 10.1107/S241431462300679X

**Published:** 2023-08-10

**Authors:** Gabrielle J. Gaskin, Robert L. LaDuca

**Affiliations:** a E-35 Holmes Hall, Michigan State University, Lyman Briggs College, 919 E. Shaw Lane, East Lansing, MI 48825, USA; Benemérita Universidad Autónoma de Puebla, México

**Keywords:** crystal structure, coordination polymer, layer, copper

## Abstract

In the title compound, {[Cu(C_10_H_8_O_4_)(C_14_H_14_N_4_O_2_)]·H_2_O}_*n*_, the Cu^II^ cations are coordinated in a square-pyramidal fashion, with *trans* pyridyl-N donor atoms from two *N*-(2-(pyridin-3-yl­amino)­eth­yl)isonicotinamide (pein) ligands in the basal plane. The other three coordination sites are taken up by carboxyl­ate O-atom donors from three different 3-(2-carb­oxy­phen­yl)propionate (cpp) ligands. The central ethyl­enedi­amine segments of the pein ligands are disordered equally over two sets of positions. {Cu_2_O_2_} rhomboid clusters are connected into [Cu(cpp)(pein)]_*n*_ (4,4) coordination polymer grids by the full span of the cpp and pein ligands. Individual layer motifs stack in an *AAA* pattern along the *a* axis by means of inter­layer hydrogen-bonding inter­actions.

## Abstract 2

A layered Cu^II^ coordination polymer with (4,4) topology was prepared by hydro­thermal methods and structurally characterized by single-crystal X-ray diffraction.

## Graphical abstract

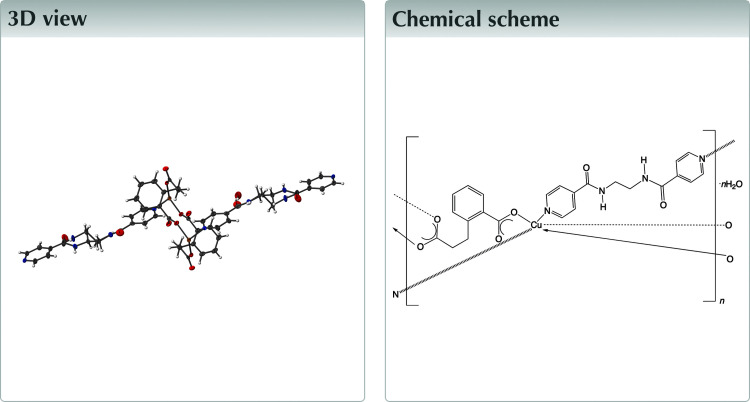

## Structure description

The title compound was isolated during an exploratory synthetic effort aiming to produce a copper coordination polymer containing both 3-(2-carb­oxy­phen­yl)propionate (cpp) and *N*-(2-(pyridin-3-yl­amino)­eth­yl)nicotinamide (pein) ligands. Our group has previously reported a chiral cobalt camphorate pein-containing coordination polymer that manifests a twofold parallel inter­penetrated looped layer topology (Przybyla *et al.*, 2019 ▸).

**Figure 1:**
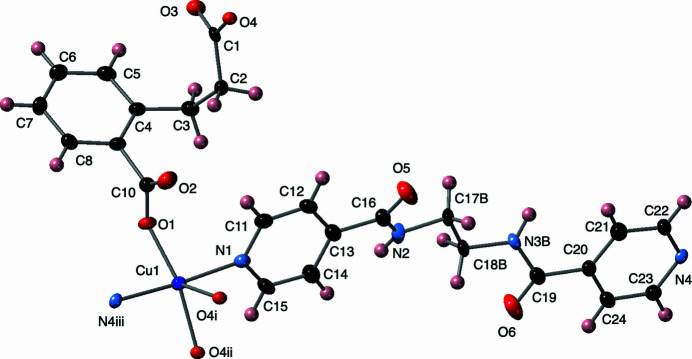
Coordination environment in the title compound with full ligand set. Displacement ellipsoids are drawn at the 50% probability level. Only one of the disordered components in pein is shown. Color code: Cu, dark blue; O, red; N, light blue; C, black; H, pink. Symmetry codes are as listed in Table 1 ▸.

The asymmetric unit of the title compound contains a divalent copper atom, a fully deprotonated cpp ligand, a pein ligand, and a water mol­ecule of crystallization. The central ethyl­enedi­amine segments of the pein ligands are disordered equally over two sets of positions. The copper atoms in the title compound display an [N_2_O_3_] square pyramidal coordination environment (Fig. 1 ▸), with *trans* pyridyl-N donor atoms from two pein ligands in the basal plane. The other two *trans* basal-plane sites are taken up by a shorter-arm carboxyl­ate O-atom donor from a cpp ligand and a longer-arm carboxyl­ate O atom donor from another cpp ligand; the apical site shows an elongated bond to the copper atom and is filled by a longer-arm carboxyl­ate O-atom donor belonging to a third cpp ligand. The trigonality factor τ is 0.289 (Addison *et al.*, 1984 ▸), indicating a significant distortion from idealized square-pyramidal geometry. Relevant bond lengths and angles within the coordination environment in the title complex are listed in Table 1 ▸.

**Figure 2:**
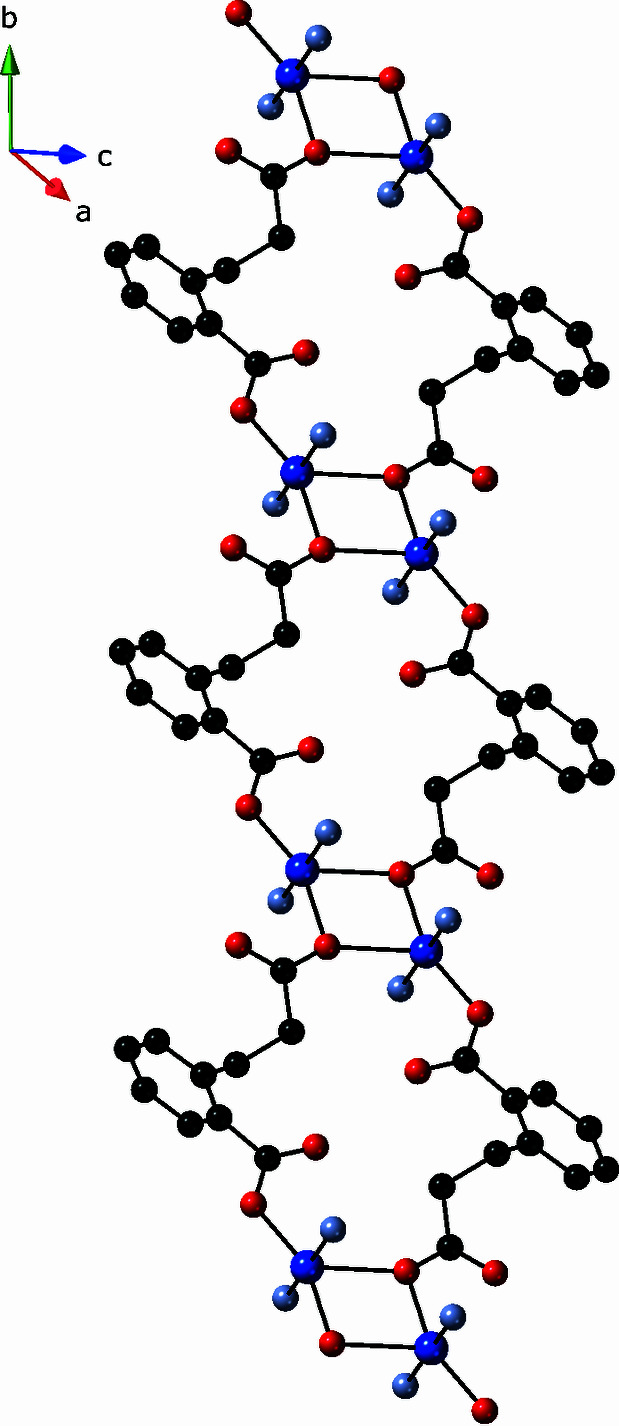
[Cu_2_(cpp)_2_]_*n*_ coordination polymer chain in the title compound, featuring [Cu_2_(O)_2_] rhomboid clusters.

**Figure 3:**
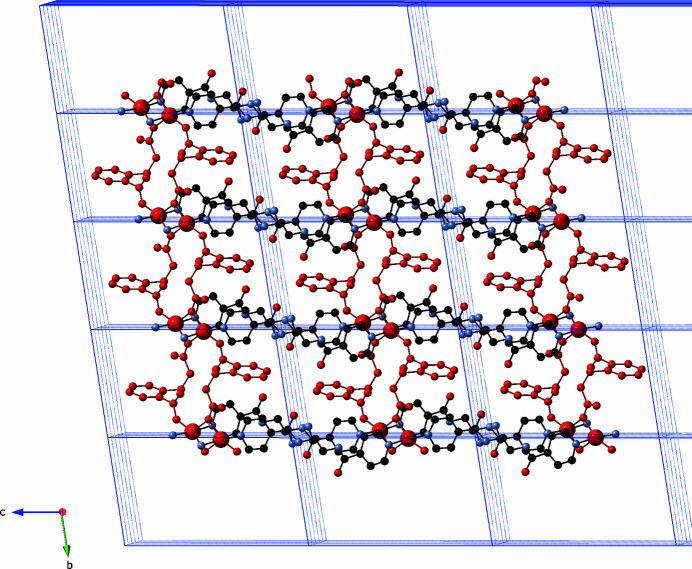
[Cu(cpp)(pein)]_*n*_ coordination polymer layer in the title compound, with [Cu_2_(cpp)_2_]_*n*_ coordination polymer chains drawn in red.

The cpp ligand in the title complex exhibits an exotridentate binding mode in which the longer carboxyl­ate arm bridges two Cu^II^ atoms while the shorter carboxyl­ate group acts as a monodentate donor to a single Cu^II^ atom. A single carboxyl­ate oxygen donor atom from the longer cpp terminus bridges two copper atoms, connecting to a basal site on one and an apical site on the other. Pairs of these inter­actions construct {Cu_2_O_2_} rhomboid dimeric units with a Cu⋯Cu distance of 3.50 (1) Å and inter­nal angles of 103.67 (7)° (Cu—O—Cu) and 76.33 (8)° (O—Cu—O). The full span of the cpp ligands connects the {Cu_2_O_2_} dimeric units into [Cu_2_(cpp)_2_]_*n*_ coordination polymer chains that are oriented parallel to the *b* axis (Fig. 2 ▸). The chains are pillared into [Cu(cpp)(pein)]_*n*_ coordination polymer layers arranged parallel to (101) (Fig. 3 ▸) by pairs of pein ligands that span a Cu⋯Cu distance of 17.64 (1) Å. The pairs of pein ligands inter­act *via* π–π stacking between their pyridyl rings (approximate distance between centroids: 3.6 Å).

**Figure 4:**
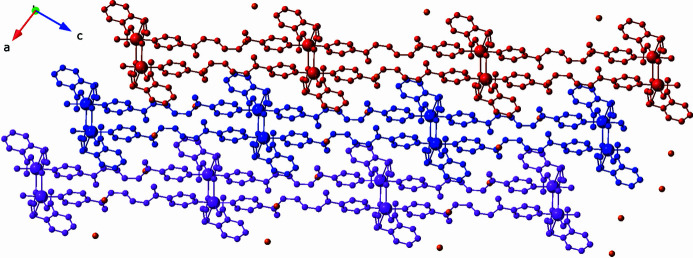
*AAA* stacking of [Cu(cpp)(pein)]_*n*_ coordination polymer layers along the *a*axis. O atoms belonging to unligated water mol­ecules of crystallization are depicted as orange spheres.

Supra­molecular inter­actions are present in the crystal structure. Individual [Cu(cpp)(pein)]_*n*_ coordination polymer layers stack in an *AAA* pattern along the *a*-axis direction (Blatov *et al.*, 2014 ▸; Fig. 4 ▸) by means of inter­layer hydrogen-bonding donation between pein N—H groups and bound shorter-arm cpp carboxyl­ate-O atoms. Isolated water mol­ecules of crystallization are located in small pockets in the inter­lamellar regions, held to the coordination polymer layers by hydrogen-bonding donation to pein C=O groups. Details regarding the hydrogen bonding in the title compound are listed in Table 2 ▸.

## Synthesis and crystallization

Cu(NO_3_)_2_·2.5 H_2_O (87 mg, 0.37 mmol), 3-(2-carb­oxy­phen­yl)propionic acid (cppH_2_, 73 mg, 0.37 mmol), *N*-(2-(pyridin-3-yl­amino)­eth­yl)isonicotinamide (pein, 100 mg, 0.37 mmol) and 0.75 ml of a 1.0 *M* NaOH solution were placed into 10 ml of distilled water in a Teflon-lined acid digestion bomb. The bomb was sealed and heated in an oven at 373 K for 48 h, and then cooled slowly to 273 K. Blue crystals of the title complex were obtained in 77% yield.

## Refinement

Crystal data, data collection and structure refinement details are summarized in Table 3 ▸. Atoms C17, C18 and N3 in the pein ligand are disordered over two sites (labeled *A* and *B*), and were refined with site occupancies fixed to 1/2. This part was refined with free coordinates, with no restraints applied to the geometry or to displacement parameters.

## Supplementary Material

Crystal structure: contains datablock(s) I, global. DOI: 10.1107/S241431462300679X/bh4076sup1.cif

Structure factors: contains datablock(s) I. DOI: 10.1107/S241431462300679X/bh4076Isup2.hkl

CCDC reference: 1979280

Additional supporting information:  crystallographic information; 3D view; checkCIF report

## Figures and Tables

**Table 1:** Selected geometric parameters (Å, °)

Cu1—O1	1.9754 (18)	Cu1—N1	2.010 (2)
Cu1—O4^i^	2.4322 (19)	Cu1—N4^iii^	2.033 (2)
Cu1—O4^ii^	2.0007 (18)		
			
O1—Cu1—O4^ii^	156.66 (8)	O4^ii^—Cu1—N1	92.95 (8)
O1—Cu1—O4^i^	126.81 (7)	O4^ii^—Cu1—N4^iii^	91.12 (8)
O1—Cu1—N1	91.16 (8)	N1—Cu1—O4^i^	87.84 (8)
O1—Cu1—N4^iii^	86.83 (8)	N1—Cu1—N4^iii^	173.98 (9)
O4^ii^—Cu1—O4^i^	76.33 (8)	N4^iii^—Cu1—O4^i^	88.83 (8)

Symmetry codes: (i) 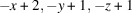 ; (ii) 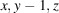 ; (iii) 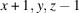 .

**Table 2:** Hydrogen-bond geometry (Å, °)

*D*—H⋯*A*	*D*—H	H⋯*A*	*D*⋯*A*	*D*—H⋯*A*
N2—H2*B*⋯O1^iv^	0.88	2.12	2.959 (3)	160
N3*B*—H3*BA*⋯O1*W* ^v^	0.88	1.99	2.836 (5)	160
O1*W*—H1*WA*⋯O5	0.87	1.92	2.781 (3)	172

Symmetry codes: (iv) 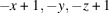 ; (v) 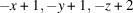 .

**Table 3:** Experimental details

Crystal data	
Chemical formula	[Cu(C_10_H_8_O_4_)(C_14_H_14_N_4_O_2_)]·H_2_O
*M*_r_	544.01
Crystal system, space group	Triclinic, *P* 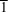
Temperature (K)	173
*a*, *b*, *c* (Å)	9.1138 (8), 9.4045 (8), 14.4758 (13)
α, β, γ (°)	97.300 (1), 94.045 (1), 114.149 (1)
*V* (Å^3^)	1112.63 (17)
*Z*	2
Radiation type	Mo *K*α
μ (mm^−1^)	1.04
Crystal size (mm)	0.15 × 0.13 × 0.10

Data collection	
Diffractometer	Bruker APEXII CCD
Absorption correction	Multi-scan (*SADABS*; Krause *et al.*, 2015 ▸)
*T*_min_, *T*_max_	0.676, 0.745
No. of measured, independent and observed [*I* > 2σ(*I*)] reflections	18015, 4077, 3323
*R*_int_	0.055
(sin θ/λ)_max_ (Å^−1^)	0.604

Refinement	
*R*[*F* ^2^ > 2σ(*F* ^2^)], *wR*(*F* ^2^), *S*	0.039, 0.092, 1.05
No. of reflections	4077
No. of parameters	355
H-atom treatment	H-atom parameters constrained
Δρ_max_, Δρ_min_ (e Å^−3^)	0.52, −0.30

Computer programs: *COSMO* (Bruker, 2009 ▸), *SAINT* (Bruker, 2013 ▸), *SHELXT2018/2* (Sheldrick, 2015*a* ▸), *SHELXL2018/3* (Sheldrick, 2015*b* ▸), *CrystalMaker X* (Palmer, 2020 ▸), and *OLEX2* (Dolomanov *et al.*, 2009 ▸).

## full crystallographic data

### Crystal data

**Table:**

[Cu(C_10_H_8_O_4_)(C_14_H_14_N_4_O_2_)]·H_2_O	*Z* = 2
*M**_r_* = 544.01	*F*(000) = 562
Triclinic, *P*1	*D*_x_ = 1.624 Mg m^−^^3^
*a* = 9.1138 (8) Å	Mo *K*α radiation, λ = 0.71073 Å
*b* = 9.4045 (8) Å	Cell parameters from 6936 reflections
*c* = 14.4758 (13) Å	θ = 2.4–25.3°
α = 97.300 (1)°	µ = 1.04 mm^−^^1^
β = 94.045 (1)°	*T* = 173 K
γ = 114.149 (1)°	Block, blue
*V* = 1112.63 (17) Å^3^	0.15 × 0.13 × 0.10 mm

### Data collection

**Table:**

Bruker APEXII CCD diffractometer	3323 reflections with *I* > 2σ(*I*)
φ and ω scans	*R*_int_ = 0.055
Absorption correction: multi-scan (SADABS; Krause *et al.*, 2015)	θ_max_ = 25.4°, θ_min_ = 1.4°
*T*_min_ = 0.676, *T*_max_ = 0.745	*h* = −10→10
18015 measured reflections	*k* = −11→11
4077 independent reflections	*l* = −17→17

### Refinement

**Table:**

Refinement on *F*^2^	Primary atom site location: dual
Least-squares matrix: full	Secondary atom site location: difference Fourier map
*R*[*F*^2^ > 2σ(*F*^2^)] = 0.039	Hydrogen site location: mixed
*wR*(*F*^2^) = 0.092	H-atom parameters constrained
*S* = 1.05	*w* = 1/[σ^2^(*F*_o_^2^) + (0.0352*P*)^2^ + 0.9143*P*] where *P* = (*F*_o_^2^ + 2*F*_c_^2^)/3
4077 reflections	(Δ/σ)_max_ < 0.001
355 parameters	Δρ_max_ = 0.52 e Å^−^^3^
0 restraints	Δρ_min_ = −0.30 e Å^−^^3^
0 constraints	

### Fractional atomic coordinates and isotropic or equivalent isotropic displacement parameters (Å^2^)

**Table:**

	*x*	*y*	*z*	*U*_iso_*/*U*_eq_	Occ. (<1)
Cu1	0.86606 (4)	0.03522 (4)	0.42367 (2)	0.01990 (12)	
O1	0.7782 (2)	0.1460 (2)	0.34432 (13)	0.0228 (4)	
O2	0.9787 (2)	0.3862 (2)	0.39173 (14)	0.0293 (5)	
O3	0.6356 (2)	0.7371 (2)	0.38655 (14)	0.0266 (5)	
O4	0.8764 (2)	0.8502 (2)	0.47353 (12)	0.0184 (4)	
O5	0.6664 (3)	0.3509 (3)	0.83105 (16)	0.0496 (7)	
O6	0.1264 (3)	−0.1854 (3)	1.01159 (17)	0.0508 (7)	
N1	0.7650 (3)	0.0919 (3)	0.53328 (16)	0.0192 (5)	
N2	0.4476 (3)	0.1163 (3)	0.80295 (17)	0.0337 (7)	
H2A	0.402136	0.029931	0.760226	0.040*	0.5
H2B	0.386529	0.023682	0.768121	0.040*	0.5
N4	−0.0154 (3)	0.0023 (3)	1.31488 (15)	0.0192 (5)	
C1	0.7449 (3)	0.7266 (3)	0.43646 (18)	0.0179 (6)	
C2	0.7338 (3)	0.5698 (3)	0.45840 (19)	0.0209 (6)	
H2C	0.706902	0.561637	0.523154	0.025*	
H2D	0.841825	0.568788	0.456932	0.025*	
C3	0.6088 (3)	0.4244 (3)	0.39172 (19)	0.0234 (6)	
H3A	0.597505	0.328278	0.417405	0.028*	
H3B	0.502207	0.429417	0.388582	0.028*	
C4	0.6538 (3)	0.4107 (3)	0.29341 (19)	0.0212 (6)	
C5	0.5820 (4)	0.4578 (3)	0.2218 (2)	0.0283 (7)	
H5	0.501563	0.494530	0.234762	0.034*	
C6	0.6262 (4)	0.4517 (4)	0.1325 (2)	0.0329 (8)	
H6	0.574460	0.482262	0.084503	0.039*	
C7	0.7452 (4)	0.4016 (4)	0.1125 (2)	0.0345 (8)	
H7	0.777813	0.400657	0.051600	0.041*	
C8	0.8165 (4)	0.3526 (3)	0.1821 (2)	0.0284 (7)	
H8	0.897859	0.317403	0.168642	0.034*	
C9	0.7700 (3)	0.3546 (3)	0.27152 (19)	0.0214 (6)	
C10	0.8508 (3)	0.2954 (3)	0.34283 (19)	0.0213 (6)	
C11	0.8071 (4)	0.2444 (3)	0.5709 (2)	0.0241 (6)	
H11	0.879640	0.325033	0.541595	0.029*	
C12	0.7493 (4)	0.2880 (3)	0.6497 (2)	0.0258 (7)	
H12	0.782433	0.396716	0.674135	0.031*	
C13	0.6430 (3)	0.1733 (3)	0.69311 (19)	0.0229 (6)	
C14	0.5999 (3)	0.0165 (4)	0.6550 (2)	0.0257 (7)	
H14	0.528086	−0.066012	0.683360	0.031*	
C15	0.6620 (3)	−0.0186 (3)	0.5757 (2)	0.0246 (7)	
H15	0.629962	−0.126675	0.549909	0.030*	
C16	0.5861 (4)	0.2215 (4)	0.7822 (2)	0.0294 (7)	
C17A	0.3515 (11)	0.1133 (11)	0.8843 (7)	0.023 (2)	0.5
H17A	0.354723	0.219492	0.903713	0.028*	0.5
H17B	0.236819	0.037467	0.864359	0.028*	0.5
C18A	0.4206 (8)	0.0660 (8)	0.9653 (4)	0.0310 (15)	0.5
H18A	0.408005	−0.043997	0.947555	0.037*	0.5
H18B	0.537985	0.135696	0.981143	0.037*	0.5
N3A	0.3389 (7)	0.0770 (6)	1.0475 (4)	0.0291 (12)	0.5
H3AA	0.384594	0.160658	1.092118	0.035*	0.5
C17B	0.4120 (11)	0.1782 (10)	0.8929 (7)	0.022 (2)	0.5
H17C	0.372090	0.260031	0.885677	0.026*	0.5
H17D	0.508849	0.223066	0.941254	0.026*	0.5
C18B	0.2807 (8)	0.0313 (7)	0.9173 (4)	0.0205 (13)	0.5
H18C	0.183417	−0.006668	0.869700	0.025*	0.5
H18D	0.319387	−0.053069	0.915379	0.025*	0.5
N3B	0.2361 (7)	0.0618 (6)	1.0108 (3)	0.0214 (11)	0.5
H3BA	0.249314	0.157652	1.034990	0.026*	0.5
C19	0.1776 (5)	−0.0527 (4)	1.0571 (2)	0.0455 (10)	
C20	0.1198 (4)	−0.0256 (4)	1.1499 (2)	0.0271 (7)	
C21	0.1590 (4)	0.1213 (4)	1.2039 (2)	0.0307 (7)	
H21	0.232813	0.214883	1.185214	0.037*	
C22	0.0903 (4)	0.1301 (3)	1.28488 (19)	0.0238 (7)	
H22	0.118846	0.231415	1.321265	0.029*	
C23	−0.0487 (4)	−0.1394 (3)	1.2646 (2)	0.0239 (6)	
H23	−0.119759	−0.231360	1.286046	0.029*	
C24	0.0158 (4)	−0.1577 (4)	1.1828 (2)	0.0270 (7)	
H24	−0.011059	−0.260576	1.149137	0.032*	
O1W	0.7821 (3)	0.6433 (3)	0.94803 (19)	0.0512 (7)	
H1WA	0.755433	0.552414	0.912263	0.077*	
H1WB	0.886751	0.680825	0.962743	0.077*	

### Atomic displacement parameters (Å^2^)

**Table:**

	*U*^11^	*U*^22^	*U*^33^	*U*^12^	*U*^13^	*U*^23^
Cu1	0.0247 (2)	0.0197 (2)	0.01987 (19)	0.01197 (16)	0.00789 (14)	0.00749 (14)
O1	0.0260 (11)	0.0171 (10)	0.0282 (11)	0.0103 (9)	0.0042 (9)	0.0092 (8)
O2	0.0286 (12)	0.0252 (12)	0.0287 (12)	0.0069 (10)	−0.0062 (10)	0.0066 (9)
O3	0.0277 (12)	0.0248 (11)	0.0287 (11)	0.0142 (10)	−0.0027 (9)	0.0019 (9)
O4	0.0243 (11)	0.0144 (10)	0.0183 (10)	0.0092 (9)	0.0055 (8)	0.0048 (8)
O5	0.0671 (18)	0.0453 (16)	0.0350 (14)	0.0308 (14)	−0.0041 (13)	−0.0166 (12)
O6	0.0678 (18)	0.0453 (16)	0.0375 (14)	0.0246 (14)	0.0194 (13)	−0.0083 (12)
N1	0.0186 (12)	0.0189 (12)	0.0234 (12)	0.0099 (10)	0.0047 (10)	0.0070 (10)
N2	0.0466 (18)	0.0524 (18)	0.0169 (13)	0.0338 (16)	0.0121 (12)	0.0081 (12)
N4	0.0248 (13)	0.0222 (13)	0.0134 (11)	0.0118 (11)	0.0031 (10)	0.0057 (10)
C1	0.0218 (15)	0.0186 (15)	0.0151 (13)	0.0097 (13)	0.0099 (12)	0.0008 (11)
C2	0.0268 (16)	0.0185 (15)	0.0173 (14)	0.0089 (13)	0.0052 (12)	0.0037 (11)
C3	0.0240 (16)	0.0179 (15)	0.0278 (16)	0.0081 (13)	0.0046 (13)	0.0040 (12)
C4	0.0254 (16)	0.0118 (14)	0.0219 (15)	0.0051 (12)	−0.0033 (12)	0.0003 (11)
C5	0.0335 (18)	0.0210 (16)	0.0301 (17)	0.0147 (14)	−0.0056 (14)	−0.0014 (13)
C6	0.048 (2)	0.0299 (18)	0.0230 (16)	0.0217 (16)	−0.0094 (15)	0.0008 (13)
C7	0.057 (2)	0.0334 (19)	0.0167 (15)	0.0239 (17)	0.0008 (15)	0.0025 (13)
C8	0.0403 (19)	0.0265 (17)	0.0241 (16)	0.0204 (15)	0.0053 (14)	0.0014 (13)
C9	0.0264 (16)	0.0131 (14)	0.0225 (15)	0.0070 (12)	−0.0006 (12)	0.0028 (11)
C10	0.0246 (16)	0.0221 (16)	0.0211 (15)	0.0125 (13)	0.0065 (12)	0.0058 (12)
C11	0.0254 (16)	0.0182 (15)	0.0288 (16)	0.0087 (13)	0.0038 (13)	0.0069 (12)
C12	0.0333 (18)	0.0176 (15)	0.0268 (16)	0.0123 (14)	0.0002 (13)	0.0008 (12)
C13	0.0209 (15)	0.0269 (16)	0.0212 (15)	0.0131 (13)	−0.0033 (12)	−0.0022 (12)
C14	0.0218 (16)	0.0247 (16)	0.0286 (16)	0.0074 (13)	0.0092 (13)	0.0034 (13)
C15	0.0269 (17)	0.0163 (15)	0.0295 (16)	0.0077 (13)	0.0113 (13)	0.0003 (12)
C16	0.041 (2)	0.0350 (19)	0.0181 (15)	0.0245 (16)	−0.0038 (14)	−0.0023 (14)
C17A	0.026 (6)	0.025 (6)	0.023 (4)	0.012 (4)	0.013 (5)	0.008 (5)
C18A	0.039 (4)	0.037 (4)	0.021 (3)	0.016 (3)	0.015 (3)	0.011 (3)
N3A	0.039 (4)	0.026 (3)	0.018 (3)	0.008 (3)	0.016 (3)	0.003 (2)
C17B	0.029 (6)	0.019 (5)	0.015 (4)	0.006 (4)	0.014 (4)	0.005 (4)
C18B	0.026 (4)	0.021 (3)	0.013 (3)	0.009 (3)	0.005 (3)	−0.001 (3)
N3B	0.028 (3)	0.018 (3)	0.017 (3)	0.009 (2)	0.007 (2)	−0.002 (2)
C19	0.092 (3)	0.031 (2)	0.0285 (18)	0.035 (2)	0.0304 (19)	0.0125 (16)
C20	0.0428 (19)	0.0262 (17)	0.0186 (15)	0.0191 (15)	0.0103 (14)	0.0064 (13)
C21	0.042 (2)	0.0238 (17)	0.0277 (17)	0.0118 (15)	0.0160 (14)	0.0089 (13)
C22	0.0327 (17)	0.0210 (16)	0.0183 (14)	0.0113 (14)	0.0082 (13)	0.0031 (12)
C23	0.0295 (17)	0.0205 (16)	0.0232 (15)	0.0106 (13)	0.0080 (13)	0.0068 (12)
C24	0.0387 (19)	0.0215 (16)	0.0211 (15)	0.0141 (14)	0.0043 (13)	−0.0003 (12)
O1W	0.0749 (19)	0.0262 (13)	0.0500 (16)	0.0187 (13)	0.0156 (15)	0.0033 (12)

### Geometric parameters (Å, º)

**Table:**

Cu1—O1	1.9754 (18)	C9—C10	1.515 (4)
Cu1—O4^i^	2.4322 (19)	C11—H11	0.9500
Cu1—O4^ii^	2.0007 (18)	C11—C12	1.375 (4)
Cu1—N1	2.010 (2)	C12—H12	0.9500
Cu1—N4^iii^	2.033 (2)	C12—C13	1.378 (4)
O1—C10	1.289 (3)	C13—C14	1.386 (4)
O2—C10	1.227 (3)	C13—C16	1.508 (4)
O3—C1	1.234 (3)	C14—H14	0.9500
O4—C1	1.298 (3)	C14—C15	1.377 (4)
O5—C16	1.223 (4)	C15—H15	0.9500
O6—C19	1.217 (4)	C17A—H17A	0.9900
N1—C11	1.350 (3)	C17A—H17B	0.9900
N1—C15	1.336 (3)	C17A—C18A	1.493 (11)
N2—H2A	0.8800	C18A—H18A	0.9900
N2—H2B	0.8800	C18A—H18B	0.9900
N2—C16	1.331 (4)	C18A—N3A	1.462 (7)
N2—C17A	1.512 (10)	N3A—H3AA	0.8800
N2—C17B	1.470 (10)	N3A—C19	1.507 (7)
N4—C22	1.343 (4)	C17B—H17C	0.9900
N4—C23	1.338 (4)	C17B—H17D	0.9900
C1—C2	1.512 (4)	C17B—C18B	1.515 (8)
C2—H2C	0.9900	C18B—H18C	0.9900
C2—H2D	0.9900	C18B—H18D	0.9900
C2—C3	1.528 (4)	C18B—N3B	1.467 (7)
C3—H3A	0.9900	N3B—H3BA	0.8800
C3—H3B	0.9900	N3B—C19	1.288 (6)
C3—C4	1.513 (4)	C19—C20	1.506 (4)
C4—C5	1.399 (4)	C20—C21	1.389 (4)
C4—C9	1.400 (4)	C20—C24	1.386 (4)
C5—H5	0.9500	C21—H21	0.9500
C5—C6	1.382 (4)	C21—C22	1.376 (4)
C6—H6	0.9500	C22—H22	0.9500
C6—C7	1.382 (4)	C23—H23	0.9500
C7—H7	0.9500	C23—C24	1.381 (4)
C7—C8	1.386 (4)	C24—H24	0.9500
C8—H8	0.9500	O1W—H1WA	0.8703
C8—C9	1.390 (4)	O1W—H1WB	0.8701
			
O1—Cu1—O4^ii^	156.66 (8)	C11—C12—C13	119.8 (3)
O1—Cu1—O4^i^	126.81 (7)	C13—C12—H12	120.1
O1—Cu1—N1	91.16 (8)	C12—C13—C14	117.7 (3)
O1—Cu1—N4^iii^	86.83 (8)	C12—C13—C16	119.6 (3)
O4^ii^—Cu1—O4^i^	76.33 (8)	C14—C13—C16	122.6 (3)
O4^ii^—Cu1—N1	92.95 (8)	C13—C14—H14	120.3
O4^ii^—Cu1—N4^iii^	91.12 (8)	C15—C14—C13	119.5 (3)
N1—Cu1—O4^i^	87.84 (8)	C15—C14—H14	120.3
N1—Cu1—N4^iii^	173.98 (9)	N1—C15—C14	123.1 (3)
N4^iii^—Cu1—O4^i^	88.83 (8)	N1—C15—H15	118.4
C10—O1—Cu1	123.27 (18)	C14—C15—H15	118.4
Cu1^iv^—O4—Cu1^i^	103.67 (7)	O5—C16—N2	123.6 (3)
C1—O4—Cu1^i^	149.19 (17)	O5—C16—C13	120.2 (3)
C1—O4—Cu1^iv^	107.15 (16)	N2—C16—C13	116.3 (3)
C11—N1—Cu1	121.00 (18)	N2—C17A—H17A	109.6
C15—N1—Cu1	121.71 (19)	N2—C17A—H17B	109.6
C15—N1—C11	117.2 (2)	H17A—C17A—H17B	108.1
C16—N2—H2A	113.1	C18A—C17A—N2	110.2 (7)
C16—N2—H2B	125.2	C18A—C17A—H17A	109.6
C16—N2—C17A	133.7 (4)	C18A—C17A—H17B	109.6
C16—N2—C17B	109.7 (4)	C17A—C18A—H18A	109.5
C17A—N2—H2A	113.1	C17A—C18A—H18B	109.5
C17B—N2—H2B	125.2	H18A—C18A—H18B	108.0
C22—N4—Cu1^v^	118.58 (18)	N3A—C18A—C17A	110.9 (6)
C23—N4—Cu1^v^	123.67 (19)	N3A—C18A—H18A	109.5
C23—N4—C22	117.4 (2)	N3A—C18A—H18B	109.5
O3—C1—O4	121.8 (3)	C18A—N3A—H3AA	119.5
O3—C1—C2	122.2 (3)	C18A—N3A—C19	121.1 (5)
O4—C1—C2	116.0 (2)	C19—N3A—H3AA	119.5
C1—C2—H2C	108.5	N2—C17B—H17C	111.4
C1—C2—H2D	108.5	N2—C17B—H17D	111.4
C1—C2—C3	114.9 (2)	N2—C17B—C18B	102.1 (5)
H2C—C2—H2D	107.5	H17C—C17B—H17D	109.2
C3—C2—H2C	108.5	C18B—C17B—H17C	111.4
C3—C2—H2D	108.5	C18B—C17B—H17D	111.4
C2—C3—H3A	109.0	C17B—C18B—H18C	109.2
C2—C3—H3B	109.0	C17B—C18B—H18D	109.2
H3A—C3—H3B	107.8	H18C—C18B—H18D	107.9
C4—C3—C2	113.0 (2)	N3B—C18B—C17B	111.9 (6)
C4—C3—H3A	109.0	N3B—C18B—H18C	109.2
C4—C3—H3B	109.0	N3B—C18B—H18D	109.2
C5—C4—C3	120.3 (3)	C18B—N3B—H3BA	120.3
C5—C4—C9	118.0 (3)	C19—N3B—C18B	119.5 (4)
C9—C4—C3	121.7 (2)	C19—N3B—H3BA	120.3
C4—C5—H5	119.5	O6—C19—N3A	122.6 (3)
C6—C5—C4	121.1 (3)	O6—C19—N3B	116.0 (4)
C6—C5—H5	119.5	O6—C19—C20	120.3 (3)
C5—C6—H6	119.8	N3B—C19—C20	119.9 (3)
C7—C6—C5	120.5 (3)	C20—C19—N3A	113.0 (3)
C7—C6—H6	119.8	C21—C20—C19	125.3 (3)
C6—C7—H7	120.3	C24—C20—C19	117.3 (3)
C6—C7—C8	119.4 (3)	C24—C20—C21	117.4 (3)
C8—C7—H7	120.3	C20—C21—H21	120.3
C7—C8—H8	119.7	C22—C21—C20	119.5 (3)
C7—C8—C9	120.6 (3)	C22—C21—H21	120.3
C9—C8—H8	119.7	N4—C22—C21	123.1 (3)
C4—C9—C10	122.3 (2)	N4—C22—H22	118.4
C8—C9—C4	120.5 (3)	C21—C22—H22	118.4
C8—C9—C10	117.2 (3)	N4—C23—H23	118.6
O1—C10—C9	114.9 (2)	N4—C23—C24	122.8 (3)
O2—C10—O1	124.7 (3)	C24—C23—H23	118.6
O2—C10—C9	120.3 (2)	C20—C24—H24	120.1
N1—C11—H11	118.6	C23—C24—C20	119.8 (3)
N1—C11—C12	122.8 (3)	C23—C24—H24	120.1
C12—C11—H11	118.6	H1WA—O1W—H1WB	104.5
C11—C12—H12	120.1		
			
Cu1—O1—C10—O2	−2.2 (4)	C9—C4—C5—C6	−1.0 (4)
Cu1—O1—C10—C9	175.31 (17)	C11—N1—C15—C14	−0.7 (4)
Cu1^iv^—O4—C1—O3	−5.1 (3)	C11—C12—C13—C14	0.6 (4)
Cu1^i^—O4—C1—O3	175.11 (19)	C11—C12—C13—C16	177.1 (3)
Cu1^iv^—O4—C1—C2	175.05 (17)	C12—C13—C14—C15	−0.8 (4)
Cu1^i^—O4—C1—C2	−4.7 (4)	C12—C13—C16—O5	−22.3 (4)
Cu1—N1—C11—C12	−175.4 (2)	C12—C13—C16—N2	158.0 (3)
Cu1—N1—C15—C14	175.1 (2)	C13—C14—C15—N1	0.9 (5)
Cu1^v^—N4—C22—C21	−170.4 (2)	C14—C13—C16—O5	154.0 (3)
Cu1^v^—N4—C23—C24	170.2 (2)	C14—C13—C16—N2	−25.7 (4)
O3—C1—C2—C3	18.7 (4)	C15—N1—C11—C12	0.4 (4)
O4—C1—C2—C3	−161.5 (2)	C16—N2—C17A—C18A	−81.7 (8)
O6—C19—C20—C21	−174.4 (4)	C16—N2—C17B—C18B	−165.6 (5)
O6—C19—C20—C24	3.9 (5)	C16—C13—C14—C15	−177.2 (3)
N1—C11—C12—C13	−0.4 (5)	C17A—N2—C16—O5	−1.9 (7)
N2—C17A—C18A—N3A	174.7 (5)	C17A—N2—C16—C13	177.9 (6)
N2—C17B—C18B—N3B	174.4 (5)	C17A—C18A—N3A—C19	81.0 (7)
N4—C23—C24—C20	0.0 (5)	C18A—N3A—C19—O6	18.2 (8)
C1—C2—C3—C4	67.9 (3)	C18A—N3A—C19—C20	175.4 (5)
C2—C3—C4—C5	−100.4 (3)	N3A—C19—C20—C21	27.8 (5)
C2—C3—C4—C9	78.2 (3)	N3A—C19—C20—C24	−153.9 (4)
C3—C4—C5—C6	177.5 (3)	C17B—N2—C16—O5	−1.8 (6)
C3—C4—C9—C8	−175.9 (3)	C17B—N2—C16—C13	178.0 (5)
C3—C4—C9—C10	3.6 (4)	C17B—C18B—N3B—C19	−153.6 (7)
C4—C5—C6—C7	−1.3 (5)	C18B—N3B—C19—O6	−17.3 (8)
C4—C9—C10—O1	90.8 (3)	C18B—N3B—C19—C20	−175.4 (4)
C4—C9—C10—O2	−91.5 (3)	N3B—C19—C20—C21	−17.2 (6)
C5—C4—C9—C8	2.6 (4)	N3B—C19—C20—C24	161.1 (4)
C5—C4—C9—C10	−177.8 (3)	C19—C20—C21—C22	176.2 (3)
C5—C6—C7—C8	2.0 (5)	C19—C20—C24—C23	−176.1 (3)
C6—C7—C8—C9	−0.4 (5)	C20—C21—C22—N4	−0.3 (5)
C7—C8—C9—C4	−1.9 (4)	C21—C20—C24—C23	2.3 (5)
C7—C8—C9—C10	178.5 (3)	C22—N4—C23—C24	−2.5 (4)
C8—C9—C10—O1	−89.6 (3)	C23—N4—C22—C21	2.7 (4)
C8—C9—C10—O2	88.1 (3)	C24—C20—C21—C22	−2.2 (5)

Symmetry codes: (i) −*x*+2, −*y*+1, −*z*+1; (ii) *x*, *y*−1, *z*; (iii) *x*+1, *y*, *z*−1; (iv) *x*, *y*+1, *z*; (v) *x*−1, *y*, *z*+1.

### Hydrogen-bond geometry (Å, º)

**Table:**

*D*—H···*A*	*D*—H	H···*A*	*D*···*A*	*D*—H···*A*
N2—H2*B*···O1^vi^	0.88	2.12	2.959 (3)	160
N3*B*—H3*BA*···O1*W*^vii^	0.88	1.99	2.836 (5)	160
O1*W*—H1*WA*···O5	0.87	1.92	2.781 (3)	172

Symmetry codes: (vi) −*x*+1, −*y*, −*z*+1; (vii) −*x*+1, −*y*+1, −*z*+2.
